# Intravitreal Triamcinolone for Acute Branch Retinal Vein Occlusion: a Randomized Clinical Trial

**Published:** 2011-04

**Authors:** Alireza Ramezani, Morteza Entezari, Siamak Moradian, Shohreh Kadkhodaei, Homa Tabatabaei, Babak Dehsarvi, Mohammad Fatehi, Mehdi Yaseri

**Affiliations:** Ophthalmic Research Center, Shahid Beheshti University of Medical Sciences, Tehran, Iran

**Keywords:** Branch Retinal Vein Occlusion, Central Macular Thickness, Intraocular Pressure, Intravitreal Triamcinolone, Macular Edema, Neovascularization

## Abstract

**Purpose:**

To evaluate the therapeutic effect of intravitreal triamcinolone (IVT) injection for recent branch retinal vein occlusion (BRVO).

**Methods:**

In a randomized controlled clinical trial, 30 phakic eyes with recent (less than 10 weeks’ duration) BRVO were assigned to two groups. The treatment group (16 eyes) received 4 mg IVT and the control group (14 eyes) received subconjunctival sham injections. Changes in visual acuity (VA) were the main outcome measure.

**Results:**

VA and central macular thickness (CMT) changes were not significantly different between the study groups at any time point. Within group analysis showed significant VA improvement from baseline in the IVT group up to three months (P < 0.05); the amount of this change was −0.53 ± 0.46, −0.37 ± 0.50, −0.46 ± 0.50, and −0.29 ± 0.45 logMAR at 1, 2, 3, and 4 months, respectively. Corresponding VA improvements in the control group were −0.20 ± 0.37, −0.11 ± 0.46, −0.25 ± 0.58, and −0.05 ± 0.50 logMAR (all P values > 0.05). Significant reduction in CMT was noticed only in the treatment group (−172 ± 202 μm, P = 0.029) and at 4 months. Ocular hypertension occurred in 4 (25%) and 2 (14.3%) eyes in the IVT and control groups, respectively.

**Conclusion:**

A single IVT injection had a non-significant beneficial effect on VA and CMT in acute BRVO as compared to the natural history of the condition. The 3-month deferred treatment protocol advocated by the Branch Vein Occlusion Study Group may be a safer option than IVT injection considering its potential side effects.

## INTRODUCTION

Branch retinal vein occlusion (BRVO) has a prevalence of 0.6%^1^ to 1.6%^2^ and is the second most common type of retinal vascular abnormality following diabetic retinopathy.^3^ Visual loss following a recent BRVO may result from macular edema, foveal hemorrhage, and macular ischemia. The Branch Vein Occlusion Study (BVOS) Group demonstrated that laser photocoagulation improves visual outcomes to a significant degree in eyes with BRVO, provided that foveal vascularity is intact and presenting visual acuity (VA) is 20/40 to 20/200.^3^ Therefore, such management is limited to eyes with adequate macular perfusion and a specified range of vision. The same study suggested that therapy should be delayed for at least three months to permit maximum spontaneous resolution of retinal edema and hemorrhage. On the other hand, reduction of edema early after vein occlusion until restoration of collaterals seems to be of great importance in preventing permanent macular damage in these eyes,^4^,^5^ therefore postponing therapy for three months may adversely affect the outcomes of any intervention.

Administration of intravitreal or retrobulbar corticosteroids for treatment of macular edema secondary to retinal vascular disorders has gained popularity in recent years.^5^–^9^ Many studies have shown improvement in VA and macular edema in eyes with BRVO following intravitreal triamcinolone (IVT) injections.^10^–^21^ Most of them, however, have been retrospective case reports or small case series with no control group, making it difficult to distinguish whether the observed outcomes represent the natural history of the condition or a true response to therapy. The Standard Care vs. Corticosteroid for Retinal Vein Occlusion (SCORE) study^22^ undertook a multicenter randomized clinical trial to compare the safety and efficacy of 1 mg and 4 mg preservative-free IVT with that of grid macular photocoagulation (MPC). The study demonstrated no difference in VA among the study groups at 12 months. It is noteworthy that this study did not exclude eyes with old BRVO.

To the best of our knowledge, no prospective randomized clinical trial has been published to date comparing the effect of IVT with no treatment in recent-onset BRVO. We conducted this randomized controlled clinical trial to determine the outcomes of therapy with IVT and to compare them with the natural course of acute BRVO.

## METHODS

This clinical trial was approved by the Review Board/Ethics Committee of the Ophthalmic Research Center. The study protocol and its goals and limitations were explained to all participants before enrollment and informed consent was obtained from each patient.

All eyes suffering from BRVO of less than 10 weeks’ duration were considered for enrollment. Exclusion criteria consisted of monocularity, previous intraocular surgery or laser therapy, VA ≥ 20/40, glaucoma or ocular hypertension, significant media opacity, pre-existing iris or retinal neovascularization, concomitant arterial occlusion, signs of chronicity such as presence of cilioretinal and/or retinal shunt vessels, coexisting retinal disorders, and non-compliance.

The main outcome measure was best-corrected logarithm of the minimum angle of resolution (logMAR) visual acuity. Secondary outcomes included central macular thickness (CMT), intraocular pressure (IOP), and the appearance of the iris and/or retinal neovascularization. Central macular thickness was determined by optical coherence tomography (OCT-2; Zeiss, Dublin, CA, USA).

A complete ophthalmic examination was performed in all subjects at baseline. Ancillary diagnostic tests included fluorescein angiography and optical coherence tomography (OCT). Judged by a retina specialist who was masked to the patients’ groups, BRVOs were classified as ischemic or non-ischemic based on the area of capillary non-perfusion at a cutoff value of 5 disc diameters on fluorescein angiography. The presence of cystoid macular edema was determined by a petaloid appearance on late phase angiograms.

Eligible eyes were randomly assigned to case and control groups. Under sterile conditions in the operating room, injections were performed after topical anesthesia and insertion of a lid speculum. In the case group, 0.1 cc (4 mg) triamcinolone acetonide was injected intravitreally through the superotemporal quadrant 4 mm posterior to the limbus using a 27-gauge needle. In the control eyes, a sham injection of 0.1 cc lidocaine 2% was given subconjunctivally. Ophthalmic examinations were repeated at 1, 2, 3, and 4 months. OCT mapping was repeated at 2 and 4 months. Retinal thickness was measured in the central 1 mm circle of the 3.5 mm ring centered on the fixation point.

### Statistical Analysis

We utilized mean (±SD) values to describe quantitative data and percentages for qualitative data. Chi-square or Fisher’s Exact tests were used for qualitative data. The t-test was used for between-group comparisons and paired t-test was used for comparing quantitative data within the study groups, in univariate analysis. Adjustment for multiple within-group comparisons was performed by the Dunnett method. Data were analyzed using SPSS 15 (SPSS Inc., Chicago, IL, USA). The statistical level of significance was preset at 0.05.

To be able to detect a 0.4 logMAR (about 4 Snellen lines) difference in VA improvement between the study groups, a sample size of 14 eyes in each group was required at a two sided 5% level and study power of 85%.

## RESULTS

Thirty eyes, including 16 eyes in the IVT group and 14 in the control group, of 30 patients (18 male and 12 female) with mean age of 59.6 ± 10.9 (range, 40 to 78) years, fulfilled the study criteria and completed at least two examinations over a period of 4 months. Baseline characteristics of the study groups were comparable in the two groups and are summarized in table 1.

**Figure 1:**
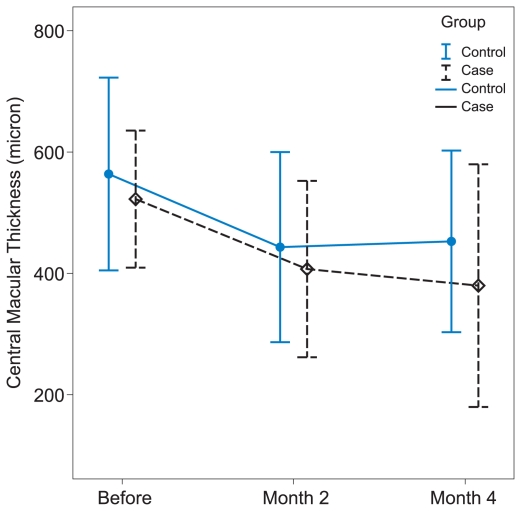
Mean corrected visual acuity (logMAR) in controls and eyes treated with 4 mg intravitreal triamcinolone at five time intervals.

Mean best-corrected VA was compared before (month 0), and 1, 2, 3 and 4 months after intervention between the study groups (Fig 1). Initial VA was comparable between the two groups. Both groups showed an improvement in VA at one month which was more pronounced in the IVT group. Better VA in the treated group was maintained up to the last follow up, however intergroup differences failed to reach a significant level at any time interval. The greatest difference in mean best-corrected VA between the groups occurred at month 1 (P = 0.087).

Differences in VA changes from baseline at each monthly visit were compared within and between the study groups (Table 2). Within the study groups, only treated eyes demonstrated significant VA changes at every visit except at month 4. However, the differences in VA changes between the groups did not reach a significant level at any time interval.

**Figure 2:**
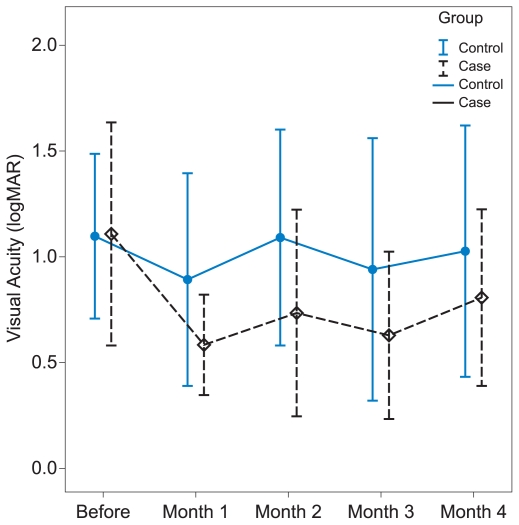
Mean central macular thickness in controls and eyes treated with 4 mg intravitreal triamcinolone at three time intervals.

Mean CMT values throughout the study are shown in figure 2. Differences in CMT changes before intervention, and 2 and 4 months afterwards were compared within and between the two groups (Table 3). Throughout the study, mean CMT was reduced in both groups. At month 2, reduction in CMT was nearly equal in the two groups; however, CMT continued to diminish further in treated eyes up to month 4 and this change was statistically significant within this subgroup. Nevertheless, differences in CMT changes between the two groups did not reach a significant level at any time interval.

The distribution of non-ischemic against ischemic BRVO was 9 vs. 7 in the treated group and 4 vs. 10 in the control group. Randomization seemed to have been unsuccessful in this regard but this was not statistically significant (P = 0.159, Fisher’s Exact test). A subgroup analysis was performed to detect the influence of this factor on VA and CMT changes (Table 4). Table 4 shows baseline values of VA and CMT and their changes up to month 4 in non-ischemic and ischemic eyes separately. Non-ischemic and ischemic eyes were significantly different in terms of mean VA and CMT at baseline. Although the degree of VA improvement and CMT reduction was greater in the ischemic subgroup, there was no significant difference between non-ischemic and ischemic eyes in terms of VA and CMT changes at month 4. This analysis demonstrated that the difference in VA and CMT changes between the groups at month 4 were significant only in the non-ischemic but not in the ischemic subgroup.

Serious injection-related complications such as vitreous hemorrhage, endophthalmitis, retinal detachment and significant cataract progression were not encountered.

During the study period, an IOP higher than 21 mmHg occurred in 4 (25%) and 2 (14.3%) eyes in IVT and control groups, respectively and none were accompanied by iris or angle neovascularization. The difference in mean IOP between the two groups reached a significant level (P = 0.035) only at the first month: 17.6 ± 5.2 mmHg vs. 13.0 ± 4.7 mmHg in the IVT and control groups, respectively.

Neovascularization of the iris occurred in three control eyes at 3 and 4 months; neovascularization of the retina appeared in one treated and one control eye at 4 months. All of these eyes underwent retinal laser photocoagulation after the study.

## DISCUSSION

In this clinical trial, although IVT injection was associated with significant visual improvement up to 3 months and reduction in CMT 4 months after intervention, these changes failed to show superiority to those observed with the natural course of recent onset BRVO. We noticed a better response to IVT therapy in eyes with non-ischemic BRVO.

Macular edema due to BRVO may resolve over a period of 6 to 24 months;^23^ however, vision may not improve because of irreversible retinal damage. The rationale behind the current trial was to initiate treatment before permanent macular damage occurs, therefore cases with more than 10 weeks’ duration were excluded.

In a retrospective study, Oh et al^4^ demonstrated that IVT is more effective in eyes with BRVO patients with less than 3 months’ duration of symptoms as compared to more chronic cases. They found VA improvement and CMT reduction at 1 month in both groups; however, this beneficial effect was maintained up to 6 months only in the early treatment group.

Scott et al^22^ conducted a multicenter, randomized clinical trial on 411 participants with BRVO (the SCORE study) and compared the safety and efficacy of 1 mg and 4 mg of preservative-free IVT with that of MPC. An increase in VA score ≥15 letters from baseline at 12 months was the main outcome measure, which was achieved in 29%, 26%, and 27% of the participants in the MPC, and 1 and 4 mg IVT groups, respectively. None of the pairwise comparisons between the three groups was significant. Considering the rates of adverse events in IVT, the authors concluded that MPC should remain the standard care for patients with macular edema secondary to BRVO. Not all of the cases in this study were naive BRVO, and not all were recent-onset disease. In our trial, however, we insisted on early intervention with IVT, before the development of irreversible macular changes, and compared it with no intervention which is the ongoing standard of care for recent BRVO based on BVOS recommendations.^3^

In a randomized clinical trial, Avitabile et al^24^ compared IVT to standard grid MPC for treatment of macular edema secondary to BRVO, diabetic retinopathy, and central retinal vein occlusion. Only 6 out of 63 eyes in this study were diagnosed with BRVO. For the entire study population, eyes receiving IVT had better VA and lower CMT values at all time points (P < 0.05). The major limitation of this study was that analysis was not conducted separately for different etiologies; therefore, no conclusion can be drawn regarding the effect of IVT on macular edema in BRVO.

Many studies on IVT for BRVO have reported a rapid but temporary improvement in VA,^10^–^18^ which has often required additional injections to maintain improvement.^11^–^15^,^18^ In our study, we found a relatively swift response of VA to IVT at one month. Although this effect decreased with time, it maintained a significant level up to month 3. The diminishing effect of treatment with IVT signifies the need for interventions with longer follow up. Compared to the control group, however, even this transient effect was not significant, which could be explained by the small sample size.

We noticed a significant decrease of 172 ± 202 microns in CMT at 4 months in IVT-treated eyes in within-group analysis which is less than that reported in other studies. For instance, in a retrospective comparative study, mean CMT decreased from 518 ± 145 to 292 ± 121 microns at three months (P = 0.001) following IVT.^25^ In another study, CMT was reported to decrease from 666 ± 291 microns at baseline to 351 ± 180 microns (P = 0.026) at 3 months.^4^ Between-group differences were not significant regarding CMT reduction which could be due to the small number of cases in the current study. The anti-edema effect of IVT along with the improvement associated with the natural course of BRVO might have resulted in further reduction in CMT after 2 months in our treatment group.

In the current trial, the amount of VA improvement and CMT reduction were more pronounced in eyes with ischemic BRVO. This may be explained by the lower VA and higher CMT at baseline in this subgroup, making a wider range of changes possible. Only in non-ischemic eyes in our study, was a significant difference detected between the subgroups in terms of both VA and CMT changes, in favor of IVT. Such an improvement following IVT has also been shown in patients with non-ischemic central retinal vein occlusion by Ip et al^26^. These investigators demonstrated a significant reduction in CMT following IVT in both non-ischemic and ischemic eyes; however, only non-ischemic patients showed statistically significant visual improvement.

On the other hand, another clinical trial revealed a significant, although transient, benefit from IVT in terms of VA and CMT in both non-ischemic and ischemic central retinal vein occlusion.^27^ It should be noted that because of the small sample size in the mentioned study, any conclusion regarding non-ischemic vs. ischemic subtypes of the disease should be made with caution.

No catastrophic injection-related complication such as retinal detachment, infectious or sterile endophthalmitis, or vitreous hemorrhage developed in our cases. In the present study, ocular hypertension developed in six eyes which was controlled with medications in all cases. The rate of steroid-induced IOP rise was lower than other studies.^14^,^27^–^30^

Regression of iris neovascularization after intravitreal injections of crystalline cortisone was reported by Jonas and associates.^31^ To our knowledge such a protective effect has not been proven in eyes with BRVO. In the current trial, neovascularization of the iris occurred in three eyes in the control group while neovascularization of the retina appeared in one eye of each study group. Due to the small number of cases with this complication, we could not reach any conclusion in this regard. To evaluate the preventive effect of IVT on iris neovascularization, a study with a larger number of cases is needed.

In the current study, sample size was calculated to allow detection of a 0.4 logMAR difference in VA change between the groups at four months. Based on the observed standard deviation in our study, the actual power of the study to detect such a difference was 60.2%. Accordingly, to be able to detect a difference as small as 0.24 logMAR, 63 and 84 cases in each group would have been required to achieve study power of 80% and 90%, respectively. However one should note that for such an intervention with its potential complications, a smaller intergroup difference may be of no clinical value. Nonetheless, small sample size and short follow up period may be considered as limitations of this study.

In summary, IVT injection was associated with slight and non-significant VA improvement one to three months after intervention as compared to the natural course of acute BRVO. Considering the temporary and non-significant effect of such therapy and its potential side effects, we recommend applying BVOS protocols for management of macular edema in eyes with BRVO, i.e., defer treatment for three months and allow time for spontaneous resolution of macular edema. However, if a more rapid short-term recovery is desired, IVT can still be considered as an option. In this case, the need for repeat injections or other interventions, such as macular photocoagulation, should be kept in mind.

Considering the promising effects of anti-vascular endothelial growth factor agents on retinal vein occlusions,^32^–^37^ further trials assessing the effects of these drugs are recommended.

## Figures and Tables

**Table 1:** Comparison of baseline characteristics in the study groups

	Treatment group		P-value
	Case	Control	
Eyes (N)	16	14	
Mean age (years)	60.1 ± 9.8	59.2 ± 12.6	0.837*
Female/male	9/7	3/11	0.057†
Mean symptom duration (weeks)	5.4 ± 3.1	6.2 ± 2.9	0.501*
Mean IOP (mmHg)	13.6 ± 3.4	12.6 ± 2.2	0.399*
Positive APD	2	3	0.490†
Non-ischemic/ischemic	9/7	4/10	0.159†
Mean VA (logMAR)	1.10 ± 0.52	1.09 ± 0.39	0.940*
Mean CMT (μ)	521 ± 113	563 ± 158	0.424*
Presence of CME‡	6 of 12	4 of 10	0.691†

* ANOVA;

† Fisher Exact test;

‡ undetermined in 8 eyes

N, number; IOP, intraocular pressure; APD, afferent pupillary defect; VA, visual acuity; CMT, central macular thickness; CME, cystoid macular edema

**Table 2:** Visual acuity changes following intravitreal triamcinolone injection versus observation in acute branch retinal vein occlusion

Month	Case		Control		P-value between groups
	
	VA changes (logMAR)†	P-value within group*	VA changes (logMAR)†	P-value within group*	
0 to 1	−0.53 ± 0.46	0.008	−0.20 ± 0.37	0.373	0.072
0 to 2	−0.37 ± 0.50	0.049	−0.11 ± 0.46	0.801	0.225
0 to 3	−0.46 ± 0.50	0.020	−0.25 ± 0.58	0.498	0.407
0 to 4	−0.29 ± 0.45	0.113	−0.05 ± 0.50	0.681	0.206

† A decrease in logMAR notation reflects an increase in VA;

* Based on Dunnett method

VA, visual acuity

**Table 3:** Central macular thickness changes following intravitreal triamcinolone injection versus observation in acute branch retinal vein occlusion

Month	Case		Control		P-value between groups
	
	CMT changes (μ)	P-value within group*	CMT changes (μ)	P-value within group*	
0 to 2	−134 ± 131	0.116	−139 ± 194	0.060	0.953
0 to 4	−172 ± 202	0.029	−93 ± 227	0.079	0.428

* Based on Dunnett method

CMT, central macular thickness

**Table 4:** Visual acuity and central macular thickness changes up to 4 months following intravitreal triamcinolone injection versus observation in acute branch retinal vein occlusion categorized by non-ischemic versus ischemic subtypes

	Mean VA at baseline (logMAR)	VA change up to month 4 (logMAR)†		P-value between groups	Mean CMT at baseline (μ)	CMT change up to month 4 (μ)		P-value between groups
	
		Case	Control			Case	Control	
Non-ischemic	0.91 ± 0.38	−0.17 ± 0.15	0.25 ± 0.44	0.027	476 ± 136	−157 ± 177	127 ± 159	0.045

Ischemic	1.24 ± 0.47	−0.59 ± 0.77	−0.14 ± 0.51	0.224	597 ± 109	−229 ± 363	−177 ± 195	0.774

P-value within group	0.048	0.128	0.262		0.015	0.287	0.041	

† A decrease in logMAR notation reflects an increase in VA

VA, visual acuity; CMT, central macular thickness
